# Organized professional response to a large-scale disaster: Earthquakes in Türkiye

**DOI:** 10.1192/j.eurpsy.2024.125

**Published:** 2024-08-27

**Authors:** K. Başar

**Affiliations:** Department of Psychiatry, Hacettepe University, Ankara, Türkiye

## Abstract

**Abstract:**

In February 2023, a series of earthquakes with high magnitudes affected 11 cities in Türkiye, a region with a population of over 13 million. With more than 50000 recorded deaths and more than 3 million survivors replaced, rescue and recovery efforts were challenging. The Psychiatric Association of Türkiye (PAT) immediately launched a “Disaster and Crisis Management,” which urgently formed and installed a program for psychosocial support and psychiatric care. The program included immediate, medium, and long-term actions. Hundreds of recruitments followed a call for volunteers for on-site and online support. An online “Earthquake and Mental Health” library was launched immediately, and a series of webinars on psychological first aid and disaster psychiatry were organized in the first two weeks. Furthermore, in three major cities, separate interactive meetings where question and answer sessions with trauma experts have been possible were held weekly with smaller groups. Almost a hundred volunteer PAT members served in the region in the first few months after the earthquake. All colleagues in the field, including those who survived the earthquakes, benefited from the resources of the PAT for their needs in housing, food, and mobilization. Starting from the first days, the PAT organized regional centers for coordination, which required financial resources and staff. The demand was high and could only be met with close collaboration with the Turkish Medical Association and the financial support obtained from international agencies, WPA, and other national psychiatric associations. The PAT started an online support system with technical support from a professional company, targeting healthcare professionals and first responders in the earthquake area. Volunteering psychiatrists provided appointment slots, rendering the system available 12 hours a day, seven days a week. With time, as the national healthcare delivery recovered, the PAT activities transformed into coordination, education, and supervision. Furthermore, the psychiatry residency training, which was interrupted due to the disaster, has been supported through a nationwide mentorship program launched by the PAT. The experience of the Psychiatric Association of Türkiye with disasters paved the way for an organized response, which was made possible through national and international solidarity.

**Disclosure of Interest:**

None Declared

